# Adequacy of Data Sources for Investigation of Tertiary Education Student’s Wellbeing in Australia: A Scoping Review

**DOI:** 10.3390/healthcare6040136

**Published:** 2018-11-26

**Authors:** Stephanie R. Partridge, Eloise Howse, Gwynnyth Llewellyn, Margaret Allman-Farinelli

**Affiliations:** 1 Nutrition and Dietetics Group, School of Life and Environmental Sciences, Charles Perkins Centre, The University of Sydney, Sydney, NSW 2006, Australia; elly.howse@sydney.edu.au (E.H.); margaret.allman-farinelli@sydney.edu.au (M.A.-F.); 2 Prevention Research Collaboration, Sydney School of Public Health, Charles Perkins Centre, The University of Sydney, Sydney, NSW 2006, Australia; 3 Westmead Applied Research Centre, Faculty of Medicine and Health, The University of Sydney, Westmead, NSW 2145, Australia; 4 Centre for Disability Research and Policy, Faculty of Health Sciences, The University of Sydney, Sydney, NSW 2006, Australia; gwynnyth.llewellyn@sydney.edu.au

**Keywords:** wellbeing, young adult, Australian, longitudinal data, higher education, policy

## Abstract

Young adulthood is a period of transition, which for many includes higher education. Higher education is associated with specific risks to wellbeing. Understanding the available data on wellbeing in this group may help inform the future collection of data to inform policy and practice in the sector. This scoping review aimed to identify the availability of data sources on the wellbeing of the Australian young adult population who are attending tertiary education. Using the methods of Arksey and O’Malley, data from three primary sources, i.e., Australian Bureau of Statistics, Australian Institute of Health and Welfare and relevant longitudinal studies, were identified. Data sources were screened and coded, and relevant information was extracted. Key data for eight areas related to wellbeing, namely, family and community, health, education and training, work, economic wellbeing, housing, crime and justice, and culture and leisure sources were identified. Forty individual data sets from 16 surveys and six active longitudinal studies were identified. Two data sets contained seven of the areas of wellbeing, of which one was specific to young adults in tertiary education, while the other survey was not limited to young adults. Both data sets lacked information concerning crime and justice variables, which have recently been identified as being of major concern among Australian university students. We recommend that government policy address the collection of a comprehensive data set encompassing each of the eight areas of wellbeing to inform future policy and practice.

## 1. Introduction

The transition from adolescence to young adulthood, broadly referred to as ages 18–35 years, is beset with challenges which impact young adults’ wellbeing. By contrast with adolescents, young adults today have reached physical maturity, yet have not established an adult life [1]. Young adulthood is characterized by instability and uncertainty, often as a result of the transitions from secondary education to work or further education or both [1]. Young adults transitioning to tertiary education, namely, higher education and vocational education training, often face financial insecurity and worse mental and physical health than during their adolescence [1,2,3]. For example, recent data indicates that 80% of Australian young adults attending higher education earn less than $AU400/week (minimum wage $AU695/week [4]), and two-thirds of 16–25 year-olds rate their mental health as poor to fair [2]. Moreover, studies have shown higher education students gain on average 3.4 kg in the first year of higher education due to changing dietary patterns and declining levels of physical activity [5].

Young adults account for the highest proportion of the population in tertiary education, which in Australia is delivered by two main institutions: Australian universities (higher education) and Technical and Further Education (TAFE; vocational education training). Higher education in Australia is that offered by a university or other higher education institution, leading to the award of a degree or higher-level qualification. Vocational education training provides people with occupational or work-related knowledge and skills, and includes programs which provide the basis for subsequent vocational programs. Vocational education training is generally provided by TAFEs and Registered Training Organizations (RTOs) [6].

In 2015, Australia had 40 universities and 53 vocational training institutes, enrolling over 1.4 million higher education students [7] and 4.2 million vocational education training students [6]. In 2016, of young adults aged 15–19 years, 83% participated in secondary or tertiary education [8]. Participation declined with age, with 45% of 20–24 year-olds and 16% of 25–34 year-olds enrolled in secondary or tertiary education [8].

Tertiary education institutions provide social, economic, and intellectual resources, which have the potential to generate benefits on a national and international scale. In 2016, people with post-secondary school education were more likely to be employed, with over 75% of persons with either a bachelor degree or above, an advanced diploma or diploma, or a certificate III or IV being employed [8]. Data from 2014 found higher education graduates alone added an estimated $140 billion to Australian gross domestic product, due to higher labor force participation, employment, and productivity [7]. Moreover, tertiary education students increase social capital by taking up leadership roles, engaging with professional associations, and interacting with their local communities [9]. Optimizing the wellbeing of young adults enrolled in tertiary education is of major significance for Australian society.

Tertiary education providers have recognized the need to optimize student experience and improve student outcomes through the promotion of wellbeing. Providing such support to students will help them assume adult roles and adopt lifestyle habits that will positively impact their wellbeing and that of their future progeny and society. Despite this recent investment into strategies to promote student wellbeing in tertiary education, little is known about what Australian data already exists on the wellbeing of young adults participating in tertiary education. Young adults are less likely to interact with health services than children or older adults, yet data from health services is commonly used to inform policy and treatment [10,11]. Thus, the collection of data via direct surveys of young adults becomes an important source of information concerning their wellbeing.

The purpose of this research was to conduct a scoping review to identify the range of available data on young adults in Australia with relevant items on wellbeing and tertiary education participation. Specifically, we aimed to: firstly, identify whole-of-population, representative sample surveys and longitudinal cohorts including items on the wellbeing of young adults attending tertiary education settings; and secondly, assess the data available across areas of concern related to wellbeing to form recommendations for policy and/or concerning routine collection of complete data capture in one survey. This would enable ongoing monitoring of young adult’s wellbeing during tertiary education, and could be used in formulating both programs and policy, as well as a way to monitor and evaluate the impact of the programs and policy introduced.

## 2. Materials and Methods

We carried out a scoping review for data sources following the methodology outlined by Arksey & O’Malley [12]. The overview for the methodological framework for this review is summarized in Table 1 and described below. This scoping review was guided by the primary question: “What quantitative data are available on the wellbeing of the Australian young adult population who are attending tertiary educational institutions?”

Between 1 November 2016 and 1 April 2017, we identified all relevant quantitative data on the wellbeing of the Australian young adult population. For inclusion, (i) data sources were required to include data specifically from young adults between the ages of 18 and 35 years, or data from representative population sample that specified age; and (ii) data sources were required to include a variable to determine at the time of data collection the type of tertiary education institution attending, if any. Quantitative data collected from the population (census) or representative samples of populations [13] and longitudinal data [14] were considered for inclusion. Data were identified from three primary sources, namely, the Australian Bureau of Statistics (ABS), the Australian Institute of Health and Welfare (AIHW), and relevant Australian longitudinal data sources. Longitudinal data were first identified from the National Centre for Longitudinal Data [15]. Additional hand searching was undertaken to identify other active Australian longitudinal data studies.

A broad definition of young adulthood, from ages 18–35 years inclusive, was adapted for this scoping review [16]. This broad definition encompasses emerging adults, typically defined as being aged 18–25 years [17], as well as young adults more generally, aged 18–35 years. This definition was also selected as 45% of 20 to 24 year-olds and 16% of 25 to 34 year-olds are enrolled in tertiary education [8]. A data source was considered for inclusion if the data was collected between 1999 and 2017, as young adults aged 35 years in 2016/2017 were aged 18 years in 1999/2000. Longitudinal data sources were considered for inclusion if a cohort was born after 1981, and at least one wave of data collection occurred between 1999 and 2017.

For the purposes of this review, the term ‘tertiary education’ was used to capture both higher education and vocational education training. A data source was included if a variable was present to distinguish the type of tertiary education institution attended at time of data collection, including higher education institutions and vocational education training or neither.

There is no universally agreed definition of wellbeing. For this scoping review, wellbeing was broadly defined as ‘a state of health or sufficiency in all areas of life’ based on ABS framework for Australian social statistics. The framework identified eight broad areas of concern of an individual’s life which contribute to their wellbeing [18]. The eight areas are: family and community, health, education and training, work, economic wellbeing, housing, crime and justice, and culture and leisure. A brief definition of each area is provided in the footnote of Table 1. The areas are varied to inform specific social issues of interest for stakeholders, including researchers, policy makers, welfare providers, and other community groups and members [18]. The ABS defines social issues as matters of concern to the government and community, which reflect aspects of society where stakeholders want to, and can, do something about them [18]. To be as comprehensive as possible, any data source that included at least one of the eight areas of concern related to wellbeing was considered for inclusion.

A list of all micro data sets available from the ABS, data cube sets AIHW, and identified longitudinal data was collated by one reviewer (SRP). To identify data sources included in the review, the same reviewer assessed data sources according to the inclusion criteria shown in Table 1 for each micro data set, data cube set, and longitudinal data source. To answer the inclusion criteria, any available online data item lists, questionnaires, related information, and explanatory notes were consulted and reviewed. An additional inclusion criterion was only relevant for the AIHW data, as administrative and minimum data sets could be excluded by title.

Details on the data sources meeting the inclusion criteria were extracted to data charting tables (Supplementary File). Information extracted included the data type, associated micro-data, aim of the data collection, how the data was collected, year(s) data were collected, geographical coverage, sample size, and data available on the social issues in each of the eight areas defined by the ABS as comprising wellbeing.

## 3. Results

**Figure 1:**
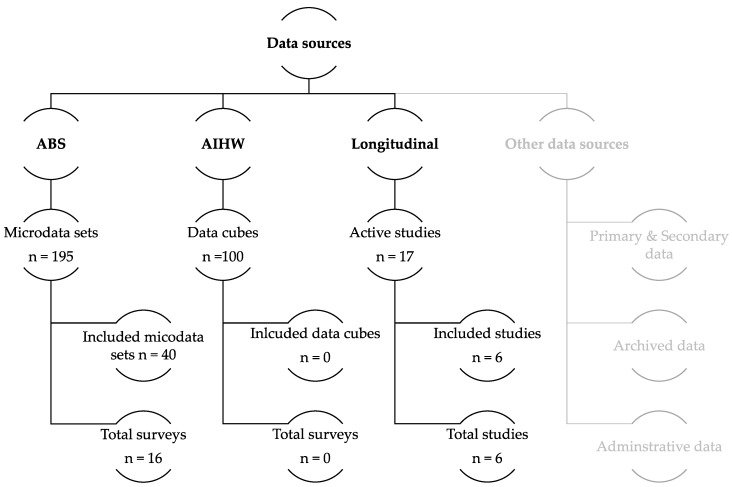
Summary of sources for identification of relevant data potentially reporting on the wellbeing of young adults living in Australia (ABS, Australian Bureau of Statistics; AIHW, Australian Institute of Health and Welfare).

Our scoping searches identified a total of 312 potential data sources (Figure 1). After initial screening, 138 data sources (122 ABS micro-data sources and 15 active longitudinal data sources) were identified for potential inclusion in the review. Of the 138 data sources, 98 ABS micro-data sources and nine active longitudinal data sources were excluded, as the data sources did not include a variable on tertiary education participation to identify if a person was attending higher education, or vocational education training, or neither. In total, 40 ABS micro-data set from 16 different surveys [19,20,21,22,23,24,25,26,27,28,29,30,31,32,33,34,35,36,37,38,39,40,41,42,43,44,45,46,47,48,49,50,51,52,53] and six active longitudinal studies [54,55,56,57,58,59] met the inclusion criteria. No data cubes from the AIHW met the inclusion criteria. Details on the 16 data sources meeting the inclusion criteria are available in Supplementary File.

A total of 38 surveys, administrated through the ABS, collected data between 1999 and 2017 on young adults aged 18–35 years and the eight areas of concern related to wellbeing (Table 2). Of these 38 surveys, 22 were excluded. A notable exclusion was the multipurpose household survey, which over the last nine years collected data, inclusive of young adults, however, did not include a variable to identify if a person was attending tertiary education. In total, 16 surveys collected data on the attendance, or not, of a higher educational institution. One of the 16 surveys was a whole of population survey (Census of Population and Housing) [19,20,21] and the remaining collected data from representative samples from the whole of Australia (for geographical coverage see Supplementary File). Three surveys collected data from a specific population sub-group (Aboriginal and Torres Strait Islander people). The remaining 12 were surveys of nationally-representative samples of Australian adults. Data sources were identified from 1999 to 2016.

A total of 15 longitudinal studies collected data between 1999 and 2017 inclusive of wellbeing-related variables in young adults aged 18–35 years (Table 3). Of these 15 studies, 6 collected data on the higher educational institution attendance [54,55,56,57,58,59]. Two were collected from representative samples of the Australian population in Victoria [55] and Australian Capital Territory and surrounds [59], and the remaining surveys collected data from representative samples from the whole of Australia. Three of the longitudinal studies began waves of data collection in 1999 or prior, and all remain active to date [55,56,59]. The remaining three began collection in 2001 [54], 2006 [57], and 2008 [58]. One study had completed data collection (from 2008 and 2010) [58]. Waves of data collection ranged from 2 to 16, with 1 to 3 cohorts. We located a website titled ‘The Australian Longitudinal Study of University Student Health and Wellbeing’ [60] that invited young adults in tertiary education to complete a questionnaire, but currently it is not a source of any data. Thus, it was excluded. The Longitudinal Surveys of Australian Youth was the only data source identified with the specific aim of tracking young adults following secondary school completion [56].

Most survey data (n = 16) were collected via face-to-face interviews, with four of these supplemented with telephone or online surveys (Supplementary File). The remaining data were collected via paper-, telephone- or web-based surveys. Five data sources are health focused (Australian Aboriginal and Torres Strait Islander Health Survey, National Aboriginal and Torres Strait Islander Health Survey, Australian Health Survey, Personality & Total Health Through Life, Australian Temperament Project), four about economic resources (Household Energy Consumption Survey, Household Expenditure Survey and Survey of Income and Housing, Household Expenditure Survey, Survey of Income and Housing), education and training (Survey of Education and Training, Survey of Education, Training and Information Technology, Adult Literacy and Life Skills Survey, Vocational Education and Training in Schools), and social issues (General Social Survey, National Aboriginal and Torres Strait Islander Social Survey, Household, Income and Labour Dynamics in Australia Survey, Longitudinal Surveys of Australian Youth); two address work (Survey of Employment Arrangements, Retirement and Superannuation, Longitudinal Labour Force Survey) and population demographic characteristics (Census of Population and Housing, Australian Census Longitudinal Dataset), and one is about family (Family Characteristics Survey) (See Supplementary File for specific details on aims of the data collection).

Data on family and community were collected from 13 surveys and from all (n = 6) longitudinal studies. The main data captured for family and community issues were the changing nature of the family (n = 16 data sources), care and support (n = 13 data sources), and voluntary work (n = 8 data sources).

Health data were collected from a total of 12 data sources (8 surveys and 4 longitudinal studies), which addressed national health priority areas (n = 9 data sources), health promotion and prevention of disease (n = 8 data sources), and socioeconomic inequalities (n = 6 data sources). Limited data on health costs and financing (n = 5 data sources) was identified.

All data sources (n = 22) collected type of education institution attended, current level of educational attainment, and field of educational attainment. Limited data were collected on equity in educational outcomes. Hours worked in paid employment were available from 19 data sources. Most data sources collected data on unemployment (n = 20), long-term unemployment (n = 18), and work and training (n = 15). Fourteen data sources provided data on voluntary work. Data on levels of remuneration and income inequality were available in 20 data sources. Twelve data sources provided data on access to resources.

Home ownership and housing costs were available in 14 and 16 of the data sources, respectively. Seven data sources collected data on access to housing, and six on community housing. There were few data sources including crime and justice and culture and leisure: two data sources reported on levels and trends of crime, however, no data was collected on personal safety and sexual harassment. Culture and leisure outcomes were collected in six data sources with the availability of leisure time available in six data sources.

## 4. Discussion

### 4.1. Principal Findings

Identifying the range of available evidence to understand the wellbeing of Australian young adults attending higher education settings is a crucial first step to inform policy about the gaps in current data collections for this purpose. To our knowledge, this is the first scoping review to identify Australian data sources on young adults’ wellbeing during tertiary education. Of the 40 individual Australian data source sets from 16 surveys and six active longitudinal studies, only one survey, the Longitudinal Surveys of Australian Youth, specifically targeted young adults’ wellbeing as they transition from secondary education to tertiary education or work [56]. Data on all the areas of wellbeing we assessed were captured in these current data collections except for crime and justice. The Australian Health Survey does not specifically target young adults, but also included all areas of wellbeing except Crime and Justice. This is an important omission, as social issues of crime and justice, including personal safety and sexual harassment, are increasingly important areas of concern for this age group [61].

### 4.2. Gaps in Current Sources of Data Collection

Regular collection of a comprehensive, representative data set in all indicative areas of wellbeing is imperative to inform national policies and interventions to improve young adults’ wellbeing. Two data collection surveys appear to require minimal change to their data collection fields to address all areas of wellbeing. One is administered by the Australian Bureau of Statistics with a regular commitment to data collection every four years (Australian Health Survey). This is a cross sectional survey using representative sampling, but which has limitations for tracking trends over time because it is not a true cohort and the interval between collections is more than the typical length of a bachelor’s degree. The advantage is that the wellbeing of graduates can be tracked beyond attendance at the tertiary institution. The update of the Australian Social Statistics framework in 2015 identified young adults as a vulnerable population, and recognized the importance of transitions over the life course [62], for example between secondary and tertiary education, and the impact of transitions on future wellbeing outcomes. There is evidence from other countries that transitions for young adults attending tertiary education are often different to those of young adults who do not attend tertiary education [63]. The other survey, the Longitudinal Surveys of Australian Youth, is administered by a private consulting company for the Department of Education and Training. The data is first collected at school, but after graduation, by telephone or online on an annual basis until age 25. It allows for tracking of behaviors over a ten-year period from age 15.

In the recent Australian Human Rights Commission survey of university students, it was found that 51% of all university students were sexually harassed at least once in 2016, and 7% were sexually assaulted [61]. Given the high prevalence of sexual harassment and assault in young people attending university, we recommend that it become government policy that crime and justice questions be added to these two existing, nationally-representative surveys. This would provide a cost-effective solution to comprehensively monitoring the wellbeing of young people in Australia.

There are many challenges for young adults after leaving school and entering tertiary education, including academic stresses, financial pressures, the challenges of leaving the family home, and the formation of new social networks [2,56,64]. Additional issues such as sleep deprivation and a culture involving alcohol binges when experienced with the previous challenges might all contribute to mental health problems [64] and declines in academic achievement. A student’s level of academic achievement significantly influences their future wellbeing, including their career path, income trajectory, health and quality of life [65]. As well as the negative impact of poor mental health on academic achievement, there is increasing evidence about the impact of both poor diet and insufficient physical activity on academic achievement [66,67]. A new research-initiated study, The Australian Longitudinal Study of University Student Health and Wellbeing, shows potential to provide useful data on the associations of higher education’s students mental health and academic performance [60]. However, as is the case with other representative population surveys, it needs to be the responsibility of the federal government statistical bureau to collect representative population-wide data to ensure national statistical standards are met and survey sustainability over time is achieved. This cannot necessarily be guaranteed by universities or other research institutions.

The data sources identified in this scoping review did not distinguish between Australian young adults in higher education settings and young adults from other countries who are international students studying in Australian higher education settings. These students would not be included in the LSAY unless they also attended secondary school in Australia prior to enrolment in an Australian university. In 2017, the higher education sector in Australia provided education to 564,869 international students [68]. Higher education for international students is Australia’s third largest export, and is valued at $21.8 billion per year, growing 17% from 2015 [7]. International students experience unique challenges while attending higher education in Australia, which can negatively impact their wellbeing [69]. To sustain and grow international student education in Australia, it is important to invest in studies which capture data on the challenges faced by this vulnerable group of students.

### 4.3. Strengths and Limitations of This Scoping Review

The strength of a scoping review methodology is that it allows the mapping of heterogeneous data sources and provides an overview of the available data [12]. The data synthesis in this review identifies gaps in population-wide generalizable data sources on specific areas of concern for the wellbeing of young Australian adults in tertiary education, and establishes the imperative for policy to ensure comprehensive data collection in an accessible form. Despite this being an extensive scoping, it is possible that some data sources may have been missed. As a scoping review, detailed examination of the questions in each of the eight wellbeing areas was not undertaken. Future steps are to lobby for the inclusion of crime and justice data in the Australian Health Survey and the Longitudinal Surveys of Australian youth, and for more detailed examination of the questions within each wellbeing area

## 5. Conclusions

We recommend that consideration be given to developing a more comprehensive system of data collection on the eight areas of wellbeing to enable regular reporting and monitoring over time of changes to the level of wellbeing and factors influencing such changes. Currently, relevant data is dispersed across different surveys, and therefore, is less accessible to focused investigation. In this scoping review, we have shown that there is no single comprehensive data set on all aspects of wellbeing of young adults attending tertiary education. Both the Longitudinal Surveys of Australian Youth and the Australian Health Survey collect data across many domains, but not issues such as personal safety and sexual harassment that are pertinent to young adults in tertiary education. Optimizing the wellbeing of all young adults enrolled in tertiary education, including domestic and international students, is of major significance for Australian society.

## Figures and Tables

**Table 1:** Overview of the Arksey and O’Malley methodological framework and application to the scoping review.

Arksey and O’Malley Framework Stage	Application to the Current Scoping Review
1. Identification of the research question	What quantitative data are available on the wellbeing of the Australian young adult population who are attending tertiary educational institutions?
2. Identification of relevant data sources	The identification of relevant Australian data involved the examination of all data sources available on the ABS website, AIHW website and all relevant longitudinal data sources.
3. Selection of relevant data sources	All data sources from step two were considered for inclusion using the inclusion criteria listed below.
	Is the data set relevant? (only for AIHW data)
	Was the data collected between 1999 and 2017?
	Does the data source include young adults?
	Does the data source include variables directly related to one or more of the following? (a) Family and community ^1^ (b) Health ^2^ (c) Education and training ^3^ (d) Work ^4^ (e) Economic resources ^5^ (f) Housing ^6^ (g) Crime and justice ^7^ (h) Culture and leisure ^8^
	Does the data source include a variable to identify tertiary institution currently attending?
4. Charting the data sources	A data charting form was completed for all data sources meeting the inclusion criteria (Supplementary file).
5. Collating and summarising the results	The dimensions of wellbeing defined by the ABS Framework for Australian Social Statistics were used to collate and categorize the data variables. The data were summarized, gaps identified and a way forward for policy in this area developed.

ABS, Australian Bureau of Statistics; AIHW, Australian Institute of Health and Welfare. ^1^ Support and nurture through family and community; ^2^ Freedom from disability and illness; ^3^ Realization of personal potential through education; ^4^ Satisfying and rewarding work both economic and non-economic; ^5^ Command over economic resources, enabling consumption; ^6^ Shelter, security, and privacy, through housing; ^7^ Personal safety and protection from crime; ^8^ Time for and access to cultural and leisure activities.

**Table 2:** Survey name and details of Australian Bureau of Statistics surveys collecting data on areas of concern impacting wellbeing of Australian young adults.

Survey Name	Survey Details					Areas of Concern Related to Wellbeing								
	Abbrev	Data Type	Reference Period	Collection Period 1999 to 2017	Includes 18 to 35-Year-Olds	Family and Community	Health	Education and Training	Work	Economic Resources	Housing	Crime and Justice	Culture and Leisure	Tertiary Education Setting
Australian Aboriginal and Torres Strait Islander Health Survey	AATSIHS	Sample	2012–2013	√	√	×	√	√	√	√	√	×	√	√
National Aboriginal and Torres Strait Islander Health Survey	NATSIHS	Sample	2004–2005	√	√	×	√	√	√	√	√	×	√	√
Census of Population and Housing	CPH	Population	2001, 2006, 2011	√	√	√	×	√	√	√	√	×	×	√
Survey of Education and Training	SET	Sample	2005, 2009	√	√	√	×	√	√	√	×	×	×	√
Survey of Education, Training and Information Technology	SETIT	Sample	2001	√	√	√	×	√	√	√	×	×	×	√
Survey of Employment Arrangements, Retirement and Superannuation	SEARS	Sample	2007	√	√	×	×	√	√	√	×	×	×	√
Family Characteristics Survey	FCS	Sample	2003	√	√	√	×	√	√	√	×	×	×	√
General Social Survey	GSS	Sample	2002, 2006, 2010, 2014	√	√	√	√	√	√	√	√	√	√	√
Household Energy Consumption Survey	HECS	Sample	2012	√	√	√	×	×	×	√	√	×	×	√
Household Expenditure Survey and Survey of Income and Housing	HES & SIH	Sample	2003–2004, 2009–2010	√	√	√	√	√	√	√	√	×	√	√
Household Expenditure Survey	HES	Sample	1998–1999	√	√	√	√	√	√	√	√	×	√	√
Survey of Income and Housing	SIH	Sample	1999–2000, 2000–2001, 2002–2003, 2005–2006, 2007–2008, 2011–2012, 2013–2014	√	√	√	×	√	√	√	√	×	×	√
National Aboriginal and Torres Strait Islander Social Survey	NATSISS	Sample	2002, 2008, 2014–2015	√	√	√	√	√	√	√	√	√	√	√
Australian Health Survey (National Health Survey)	AHS	Sample	2001, 2004–2005, 2007–2008, 2011–2012, 2014–2015	√	√	√	√	√	√	√	√	×	√	√
National Nutrition and Physical Activity Survey ^1^	NNPAS	Sample	2011–2012	√	√	×	√	×	×	×	×	×	√	√
National Health Measures Survey ^1^	NHMS	Sample	2011–2012	√	√	×	√	×	×	×	×	×	×	√
Adult Literacy and Life Skills Survey	ALLS	Sample	2006	√	√	×	√	√	√	√	×	×	×	√
Vocational Education and Training in Schools	VET	Sample	2006, 2011	√	√	√	×	√	√	√	√	×	×	√

^1^ Components of the Australian Health Survey (AHS).

**Table 3:** Study name and details of longitudinal studies collecting data on areas of concern impacting wellbeing of Australian young adults.

Study Name	Study Details						Areas of Concern Related to Wellbeing								
	Abbrev	Cohorts	Waves (Upper End)	Reference Period	Collection Period 1999 to 2017	Includes 18 to 35-Year-Olds	Family and Community	Health	Education and Training	Work	Economic Resources	Housing	Crime and Justice	Culture and Leisure	Tertiary Education Setting
Household, Income and Labour Dynamics in Australia Survey	HILDA	2	15	2001–present	√	√	√	√	√	√	√	√	×	×	√
Longitudinal Surveys of Australian Youth	LSAY	2	12	1995–present	√	√	√	√	√	√	√	√	×	√	√
Australian Census Longitudinal Dataset	ACLD	1	2	2006–present	√	√	√	×	√	√	√	√	×	√	√
Longitudinal Labour Force Survey	LLFS	1	8	2008–2010	√	√	√	×	√	√	√	√	×	×	√
Personality & Total Health Through Life	PATH	1	4	1999–present	√	√	√	√	√	√	×	√	×	√	√
Australian Temperament Project	ATP	3	16	1983–present	√	√	√	√	√	√	×	×	×	×	√

## Supplementary Materials

The following are available online at http://www.mdpi.com/2227-9032/6/4/136/s1, Table S1 to S22, completed data source charting tables for included surveys from the Australian Bureau of Statistics (ABS) (n = 16) and for the longitudinal studies conducted in Australia (n = 6).
