# Supplementary material for: Adequacy of Data Sources for Investigation of Tertiary Education Student’s Wellbeing in Australia: A Scoping Review
Source: Healthcare (Basel). 2018 Nov 26;6(4):136. doi: 10.3390/healthcare6040136 (PMC6315590; doi:10.3390/healthcare6040136)
Supplement: Supplementary file 1 [file healthcare-06-00136-s001.pdf]

Supplementary File

Tables S1–S22. data source charting tables for Australian Bureau of Statistics (ABS) surveys (n = 17)

Table S1. Australian Aboriginal and Torres Strait Islander Health Survey 2012 to 2013, (Supplementary File, ID ABS: 4-6).

**SURVEY CATEGORY**	**DETAIL**
**Abbreviation**	AATSIHS
**Data type**	Sample data (nationally representative sample)
**Associated micro data**	1. Core Content - Risk Factors and Selected Health Conditions, (NATSIHS & NATSIHMS) (Supp. 1 ID ABS: 4)<br>*Data series:* Expanded CURF, Table Builder<br>2. Detailed Conditions and Other Health Data (NATSIHS) (Supp. 1 ID ABS: 5)<br>*Data series:* Expanded CURF, Table Builder<br>3. Nutrition and Physical Activity (NATSINPAS) (Supp. 1 ID ABS: 6)<br>*Data sets:* Basic CURF<br>*Data series:* Expanded CURF, Table Builder
**Aim of the data collection**	• AATSIHS combines the existing NATSIHS together with two new surveys, the NATSINPAS and NATSIHMS to estimates of the prevalence of certain chronic diseases and conditions and selected behavioural risk factors, including physical activity participation and sedentary behaviour<br>• Objective measures of selected chronic diseases, nutrition status and other risk factors were collected, which can be combined with self-reported data about health status and conditions (e.g. diabetes)<br>• Health risk factors and outcomes for different population groups of interest, such as different age groups and people living in remote and non-remote areas
**How data was collected**	Collected by face-to-face interview (with a telephone follow-up for NATSINPAS)
**Year(s) data collected**	2012 to 2013
**Geographic coverage**	Whole of Australia (all states and territories) including non-remote areas and remote areas, including discrete communities
**Sample size**	*NATSIHS*<br>Total all ages, n=9317 (4240 M, 5077 F)<br>Total 20-34 years, n=1879 (738 M, 1141 F)<br>20-24 years, n=637 (267 M, 370 F)<br>25-29 years, n=644 (249 M, 395 F)<br>30-34 years, n=598 (222 M, 376 F)
	NATSINPAS<br>Total all ages, n=4109 (1814 M, 2295 F)<br>20-24 years, n=919 (394 M, 525 F)<br>25-29 years, n=961 (366 M, 595 F)<br>30-34 years, n=884 (334 M, 550 F)
	CORE (NATSIHS & NATSINPAS)<br>Total all ages, n=12947 (5809 M, 7138 F)<br>18-24 years, n=405 (162 M, 243 F)<br>25-29 years, n=334 (109 M, 225 F)<br>30-34 years, n=327 (110 M, 217 F)
**Wellbeing areas of concern**	Data available for key social issues for each area of concern
**(a) Family and community**	N/A

SURVEY CATEGORY	DETAIL
**(b) Health**	Aging of the population; promotion and prevention; national health priority areas and socioeconomic inequalities
**(c) Education and training**	Type of educational institution currently attending; level of educational attainment; field of educational attainment
**(d) Work**	Hours of work; work and training; unemployment; long term unemployment; regional issues; and voluntary work
**(e) Economic resources**	Income inequality; levels of remuneration and access to particular resources
**(f) Housing**	Home ownership; housing costs; access to housing; and community housing
**(g) Crime and justice**	N/A
**(h) Culture and leisure**	Culture and leisure outcomes; availability of leisure time and cultural rights
**Reference(s)**	1. Australian Bureau of Statistics. 4715.0.30.003 - Microdata: Australian Aboriginal and Torres Strait Islander Health Survey, Core Content - Risk Factors and Selected Health Conditions Canberra, Australia: Australian Bureau of Statistics; 2012-13 [01-04-2014]. Available from: http://bit.ly/2gWmwbH.<br>2. Australian Bureau of Statistics. 4715.0.30.001 - Microdata: Australian Aboriginal and Torres Strait Islander Health, Survey. Detailed Conditions and Other Health Data Canberra, Australia: Australian Bureau of Statistics; 2012-13 [01-04-2017]. Available from: http://bit.ly/2wUFCIp.<br>3. Australian Bureau of Statistics. 4715.0.30.002 - Microdata: Australian Aboriginal and Torres Strait Islander Health Survey, Nutrition and Physical Activity Canberra, Australia: Australian Bureau of Statistics; 2012-13 [01-04-2017]. Available from: http://bit.ly/2xpryIm.

Table S2. National Aboriginal and Torres Strait Islander Health Survey 2004 to 2005, (Supplementary File, ID ABS: 7).

SURVEY CATEGORY	DETAIL
**Abbreviation**	NATSIHS
**Data type**	Sample data (nationally representative sample)
**Associated micro data**	1. National Aboriginal and Torres Strait Islander Health Survey (NATSIHS) (Supp. 1 ID ABS: 7)<br>*Data series:* Expanded CURF
**Aim of the data collection**	• NATSIHS provides broad information about the health of Indigenous Australians, by remoteness, and at the national and state/territory levels<br>• Allows for the relationships across the health status and health related actions of Indigenous Australians to be explored<br>• Provide comparisons over time in the health of Indigenous Australians<br>• Provide comparisons with results for the non-Indigenous population from the NHS
**How data was collected**	Collected by face-to-face interview
**Year(s) data collected**	2004 to 2005
**Geographic coverage**	Whole of Australia (all states and territories)
**Sample size**	Total all ages, n= 10,439 Indigenous persons (from 5,234 households)<br>No further age break down provided
**Wellbeing areas of concern**	Data available for key social issues for each area of concern
**(a) Family and community**	N/A
**(b) Health**	Aging of the population; promotion and prevention; national health priority areas and socioeconomic inequalities
**(c) Education and training**	Type of educational institution currently attending; level of educational attainment; field of educational attainment
**(d) Work**	Hours of work; work and training; unemployment; long term unemployment; regional issues and voluntary work
**(e) Economic resources**	Income inequality; levels of remuneration; and access to particular resources
**(f) Housing**	Home ownership; housing costs; access to housing; and community housing
**(g) Crime and justice**	N/A
**(h) Culture and leisure**	Culture and leisure outcomes and availability of leisure time
**Reference(s)**	1. Australian Bureau of Statistics. 4715.0.55.002 - Technical Manual: National Aboriginal and Torres Strait Islander Health Survey Canberra, Australia: Australian Bureau of Statistics; 2004-05 [01-04-2017]. Available from: http://bit.ly/2gXu2qx.

Table S3. Census of population and housing 2001, 2006 and 2011, (Supplementary File, ID ABS: 16-18).

SURVEY CATEGORY	DETAIL
**Abbreviation**	CPH
**Data type**	Population data (census)
**Associated micro data**	1. Census of Population and Housing, 2011 (CPH, 2011) (Supp. 1 ID ABS: 16)<br>*Data sets:* Basic CURF<br>*Data series:* Expanded CURF<br>2. Census of Population and Housing, 2006 (CPH, 2011) (Supp. 1 ID ABS: 17)<br>*Data sets:* Basic CURF<br>*Data series:* Expanded CURF<br>3. Census of Population and Housing, 2001 (CPH, 2011) (Supp. 1 ID ABS: 18)<br>*Data sets:* Basic CURF<br>*Data series:* Expanded CURF
**Aim of the data collection**	• The aim of the CPH is to accurately collect data on the key characteristics of people in Australia on Census night and the dwellings in which they live, providing a comprehensive picture of Australia's people, how they live and where they reside.
**How data was collected**	Collected by the delivery and re-collection of a paper survey
**Year(s) data collected**	2001, 2006 and 2011
**Geographic coverage**	Whole of Australia (all states and territories)
**Sample size**	*CPH 2001*<br>Total all ages, n= 18,769,249 (9,270,466 M, 9,498,783 F)
	CPH 2006<br>Total all ages, n= 19,855,290 (9,799,249 M, 10,056,041 F)
	CPH 2011<br>Total all ages, n=21,507,717 (10,634,013 M, 10,873,704 F)<br>20-24 years, n=1,460,673<br>25-29 years, n=1,513,236<br>30-34 years, n=1,453,775
**Wellbeing areas of concern**	Data available for key social issues for each area of concern
**Family and community**	Changing nature of the family; care and support; distribution of care and support; voluntary work; wellbeing of regional and local area communities
**Health**	N/A
**Education and training**	Type of educational institution currently attending; level of educational attainment; field of educational attainment
**Work**	Hours of work; work and training; unemployment; and voluntary work
**Economic resources**	Income inequality; levels of remuneration
**Housing**	Home ownership; housing costs; access to housing; and community housing
**Crime and justice**	N/A
**Culture and leisure**	N/A
**Reference(s)**	1. Australian Bureau of Statistics. 2037.0.30.001 - Microdata: Census of Population and Housing, Census Sample File Canberra, Australia: Australian Bureau of Statistics; 2011 [01-04-2017]. Available from: http://bit.ly/2gV6tON.<br>2. Australian Bureau of Statistics. 2037.0 - Technical Manual: Census of Population and Housing, Census Sample File, Basic and Expanded CURF Canberra, Australia: Australian Bureau of Statistics; 2006 [01-04-2017]. Available from: http://bit.ly/2y3v1tj.<br>3. Australian Bureau of Statistics. 2037.0 - Census of Population and Housing: Household Sample File Technical Paper Canberra, Australia: Australian Bureau of Statistics; 2001 [01-04-2017]. Available from: http://bit.ly/2xw8Uyx

Table S4. Survey of Education and Training 2005 and 2009 (Supplementary File, ID ABS: 55-56).

SURVEY CATEGORY	DETAIL
**Abbreviation**	SET
**Data type**	Sample data (nationally representative sample)
**Associated micro data**	1. Education and Training 2005 (SET) (Supp. 1 ID ABS: 56)<br>*Data sets:* Basic CURF<br>*Data series:* Expanded CURF<br>2. Education and Training 2009 (SET) (Supp. 1 ID ABS: 55)<br>*Data sets:* Basic CURF<br>*Data series:* Expanded CURF
**Aim of the data collection**	• The survey collected information about educational and learning activity covering aspects such as educational attainment, participation in various types of education and training, and employment outcomes from education activity
**How data was collected**	Collected information in-person using computer assisted interviewing
**Year(s) data collected**	2005, 2006
**Geographic coverage**	Whole of Australia (all states and territories) excluding persons living in remote areas
**Sample size**	*SET, 2005*<br>Respondent records, n=27,577
	SET, 2009<br>Invited, n=16,400 private dwellings<br>Fully responding, n=11,800 households (n=23,807 persons)
**Wellbeing areas of concern**	Data available for key social issues for each area of concern
**(a) Family and community**	Changing nature of the family
**(b) Health**	N/A
**(c) Education and training**	Type of educational institution currently attending; level of educational attainment; field of educational attainment; barriers to education and training; education, training and work
**(d) Work**	Hours of work; work and training; unemployment; long term unemployment; voluntary work
**(e) Economic resources**	Income inequality; levels of remuneration
**(f) Housing**	N/A
**(g) Crime and justice**	N/A
**(h) Culture and leisure**	N/A
**Reference(s)**	1. Australian Bureau of Statistics. 6278.0.55.001 - Technical Manual: Education and Training, Basic and Expanded CURFs Canberra, Australia: Australian Bureau of Statistics; 2009 [01-04-2017]. Available from: http://bit.ly/2xfgtIM.<br>2. Australian Bureau of Statistics. 6278.0.55.003 - Technical Manual: Education and Training, Expanded CURF Canberra, Australia Australian Bureau of Statistics; 2005 [01-04-2017]. Available from: http://bit.ly/2xUF2Ik.

Table S5. Survey of Education, Training and Information Technology, 2001 (Supplementary File, ID ABS: 57).

SURVEY CATEGORY	DETAIL
**Abbreviation**	SETIT
**Data type**	Sample data (nationally representative sample)
**Associated micro data**	1. Education Training and Information Technology 2001 (Supp. 1 ID ABS: 57)<br>*Data sets:* Basic CURF
**Aim of the data collection**	• The survey collected detailed information on: socio-demographic characteristics; employment characteristics; educational qualifications obtained; recent study for educational qualifications; access to education and training; details of training courses completed; and use of information technology
**How data was collected**	Collected information via face-to-face personal interviews
**Year(s) data collected**	2001
**Geographic coverage**	Whole of Australia (all states and territories) excluding sparsely settled parts of Australia
**Sample size**	Respondent records, n = 24,377 individuals
**Wellbeing areas of concern**	Data available for key social issues for each area of concern
**(a) Family and community**	Changing nature of the family
**(b) Health**	N/A
**(c) Education and training**	Type of educational institution currently attending, level of educational attainment, field of educational attainment, barriers to education and training, education, training and work
**(d) Work**	Hours of work; work and training; unemployment; long term unemployment; voluntary work
**(e) Economic resources**	Income inequality; levels of remuneration
**(f) Housing**	N/A
**(g) Crime and justice**	N/A
**(h) Culture and leisure**	N/A
**Reference(s)**	1. Australian Bureau of Statistics. 6278.0.30.002 - Information Paper: Education, Training and Information Technology, Basic CURF Canberra, Australia: Australian Bureau of Statistics; 2001 [01-04-2017]. Available from: http://bit.ly/2xpULCL.

Table S6. Survey of Employment Arrangements, Retirement and Superannuation, 2007 (Supplementary File, ID ABS: 79).

SURVEY CATEGORY	DETAIL
**Abbreviation**	SEARS
**Data type**	Sample data (nationally representative sample)
**Associated micro data**	1. Employment Arrangements, Retirement and Superannuation (Supp. 1 ID ABS: 79)<br>*Data series:* Expanded CURF
**Aim of the data collection**	The main objective of SEARS 2007 was to produce a comprehensive range of data to describe:<br>• The diversity of employment arrangements in Australia, including the types of employment people have, aspects of job stability and flexibility, and working patterns;<br>• The working arrangements that people use, or would like to use to balance their work and caring responsibilities;<br>• Plans that people aged 45 years and over have for retirement, including transitions to retirement, expected sources of income at retirement and factors that might influence the decision to retire;<br>• Characteristics of retirement including age at retirement, reasons for retiring and retirement income; and<br>• The superannuation coverage of individuals, including superannuation contributions and account balances.
**How data was collected**	Collected information via face-to-face personal interviews
**Year(s) data collected**	2007
**Geographic coverage**	Whole of Australia (all states and territories) excluding non-private dwellings and very remote areas of Australia
**Sample size**	Fully responding households, n=13,736
**Wellbeing areas of concern**	Data available for key social issues for each area of concern
**(a) Family and community**	N/A
**(b) Health**	N/A
**(c) Education and training**	Type of educational institution currently attending; level of educational attainment; field of educational attainment
**(d) Work**	Hours of work; job security; work and training; unemployment; long term unemployment; unpaid household work and voluntary work
**(e) Economic resources**	Income inequality; levels of remuneration; retirement income; and access to particular resources
**(f) Housing**	N/A
**(g) Crime and justice**	N/A
**(h) Culture and leisure**	N/A
**Reference**	1. Australian Bureau of Statistics. 6361.0.55.002 - Employment Arrangements, Retirement and Superannuation, User Guide: Australian Bureau of Statistics; 2007 [01-04-2017]. Available from: http://bit.ly/2gWXuZU.

Table S7. Family Characteristics Survey, 2003 (Supplementary File, ID ABS: 84).

SURVEY CATEGORY	DETAIL
**Abbreviation**	FCS
**Data type**	Sample data (nationally representative sample)
**Associated micro data**	1. Family Characteristics (Supp. 1 ID ABS: 84)<br>*Data series:* Expanded CURF
**Aim of the data collection**	• The 2003 FCS collected details on household and family composition including demographic, labour force, and family structure information.<br>• A particular focus of the survey was families with children aged 0–17 years. The additional information collected for these families included: more specific details about relationships between family members (such as the presence of step parent/child relationships); whether any children had a natural parent living elsewhere and, if so, details of child support provided by non-resident parents and their contact arrangements; and, amount and main source of parental income.
**How data was collected**	Collected information via telephone (80%) with the remainder being face-to-face interviews
**Year(s) data collected**	2003
**Geographic coverage**	Whole of Australia (all states and territories) excluding non-private dwellings and very remote areas of Australia.
**Sample size**	Household records, n=24,498<br>Family records, n=26,172 (of which there are 17,480 actual families)<br>Person records, n=61,859
**Wellbeing areas of concern**	Data available for key social issues for each area of concern
**(a) Family and community**	Changing nature of the family; care and support; distribution of care and support; and voluntary work
**(b) Health**	N/A
**(c) Education and training**	Type of educational institution currently attending, level of education attainment; field of educational attainment
**(d) Work**	Hours of work, work and training; unemployment; long term unemployment; unpaid household work
**(e) Economic resources**	Income inequality; levels of remuneration
**(f) Housing**	N/A
**(g) Crime and justice**	N/A
**(h) Culture and leisure**	N/A
**Reference**	1. Australian Bureau of Statistics. 4442.0.55.002 - Family Characteristics Survey, Australia, Expanded Confidentialised Unit Record File: Technical Paper Canberra, Australia: Australian Bureau of Statistics; 2003 [01-04-2017]. Available from: http://bit.ly/2gWp7Ct.

Table S8. General Social Survey, 2002, 2006, 2010, 2014 (Supplementary File, ID ABS: 86-89).

SURVEY CATEGORY	DETAIL
**Abbreviation**	GSS
**Data type**	Sample data (nationally representative sample)
**Associated micro data**	1. General Social Survey 2014 (Supp. 1 ID ABS: 86)<br>*Data series:* Expanded CURF, Table Builder<br>2. General Social Survey 2010 (Supp. 1 ID ABS: 87)<br>*Data sets:* Basic CURF<br>*Data series:* Expanded CURF<br>3. General Social Survey 2006 (Supp. 1 ID ABS: 88)<br>*Data sets:* Basic CURF<br>*Data series:* Expanded CURF<br>4. General Social Survey 2002 (Supp. 1 ID ABS: 89)<br>*Data sets:* Basic CURF<br>*Data series:* Expanded CURF
**Aim of the data collection**	• The GSS is designed to enable analysis of the interrelationships in social circumstances and outcomes, including the exploration of multiple signs of advantage and disadvantage.<br>• The survey includes information on people's health, family relationships, social and community involvement, education, employment, income and financial stress, assets and liabilities, housing and mobility, crime and safety, transport, attendance at culture and leisure venues, and sports attendance and participation.
**How data is collected**	Collected information via face-to-face personal interviews
**How data was collected**	2002, 2006, 2010, 2014
**Geographic coverage**	Whole of Australia (all states and territories) excluding non-private dwellings and very remote areas of Australia.
**Sample size**	*GSS 2002*<br>Household records, n=~15,500 private dwellings
	GSS 2006<br>Household records, n=13,375 private dwellings
	GSS 2010<br>Household records, n=15,028 private dwellings
	GSS 2014<br>Household records, n= 12,932 private dwellings
**Wellbeing areas of concern**	Data available for key social issues for each area of concern
**(a) Family and community**	Changing nature of the family; family dysfunction; care and support; distribution of care and support and voluntary work
**(b) Health**	Promotion and prevention; national health priority areas; socioeconomic inequalities
**(c) Education and training**	Type of educational institution currently attending; level of educational attainment; field of educational attainment; barriers to education and training
**(d) Work**	Hours of work; work and training; unemployment; long term unemployment; unpaid household work; and voluntary work
**(e) Economic resources**	Income inequality; level of remuneration; access to particular services; retirement income
**(f) Housing**	Home ownership; housing costs; access to housing; community housing
**(g) Crime and justice**	Levels and trends of crime
**(h) Culture and leisure**	Culture and leisure outcomes; availability of leisure time
**Reference(s)**	1. Australian Bureau of Statistics. 4159.0.30.004 - Microdata: General Social Survey Canberra, Australia Australian Bureau of Statistics; 2014 [01-04-2017]. Available from: http://bit.ly/2xZHqOy

SURVEY CATEGORY	DETAIL
	2. Australian Bureau of Statistics. 4159.0.55.002 - General Social Survey: User Guide Canberra, Australia: Australian Bureau of Statistics; 2010 [01-04-2017]. Available from: http://bit.ly/2xZ9mSY<br>3. Australian Bureau of Statistics. 4159.0.55.002 - General Social Survey: User Guide Canberra, Australia: Australian Bureau of Statistics; 2006 [01-04-2017]. Available from: http://bit.ly/2ePD3xf.<br>4. Australian Bureau of Statistics. 4159.0.30.003 - Technical Manual: General Social Survey, Basic and Expanded CURF Canberra, Australia: Australian Bureau of Statistics; 2002 [01-04-2017]. Available from: http://bit.ly/2y3h8eD.

Table S9. Household Energy Consumption Survey, 2012 (Supplementary File, ID ABS: 90).

SURVEY CATEGORY	DETAIL
**Abbreviation**	HECS
**Data type**	Sample data (nationally representative sample)
**Associated micro data**	1. Household Energy Consumption (Supp. 1 ID ABS: 90)<br>*Data sets:* Basic CURF<br>*Data series:* Expanded CURF
**Aim of the data collection**	• Participating households where ask to complete a web-based questionnaire on their household's recent energy usage, costs and behaviours.<br>• Households where asked to volunteer for follow up questions approximately every three months following the household interview.
**How data was collected**	Web-based questionnaire
**Year(s) data collected**	2012
**Geographic coverage**	Whole of Australia (all states and territories) excluding non-private dwellings and very remote areas of Australia.
**Sample size**	Household records, n=11,978 (from households who participated in the second half of the 2011-12 Survey of Income and Housing)
**Wellbeing areas of concern**	Data available for key social issues for each area of concern
**(a) Family and community**	Changing nature of the family; care and support and distribution of care and support
**(b) Health**	N/A
**(c) Education and training**	Type of educational institution currently attending; level of educational attainment and field of educational attainment
**(d) Work**	N/A
**(e) Economic resources**	Income inequality; levels of remuneration and retirement income
**(f) Housing**	Home ownership and housing costs
**(g) Crime and justice**	N/A
**(h) Culture and leisure**	N/A
**Reference**	1. Australian Bureau of Statistics. 4670.0.30.001 - Microdata: Household Energy Consumption Canberra, Australia: Australian Bureau of Statistics; 2012 [01-04-2017]. Available from: http://bit.ly/2h1V6kx.

Table S10. Household Expenditure Survey and Survey of Income and Housing, 2003-04, 2009-10 (Supplementary File, ID ABS: 94-95).

SURVEY CATEGORY	DETAIL
**Abbreviation**	HES & SIH
**Data type**	Sample data (nationally representative sample)
**Associated micro data**	1. Household Expenditure Survey and Survey of Income and Housing, including Fiscal Incidence Study (Supp. 1 ID ABS: 94)<br>*Data sets:* Basic CURF<br>*Data series:* Expanded CURF<br>2. Household Expenditure Survey and Survey of Income and Housing, including Fiscal Incidence Study (Supp. 1 ID ABS: 95)<br>*Data sets:* Basic CURF<br>*Data series:* Expanded CURF
**Aim of the data collection**	• The combined SIH and HES samples were designed to produce reliable estimates for broad aggregates of total income and total expenditure for households resident in private dwellings for Australia, for each state and for the capital cities in each state and territory
**How data was collected**	Collected information via face-to-face personal interviews
**Year(s) data collected**	2003-04 and 2009-10
**Geographic coverage**	Whole of Australia (all states and territories) excluding non-private dwellings and very remote areas of Australia.
**Sample size**	*HES & SIH 2003-04*<br>Household records, n=11,361 private dwellings
	HES & SIH 2009-10<br>Household records, n=14,864 private dwellings
**Wellbeing areas of concern**	Data available for key social issues for each area of concern
**(a) Family and community**	Changing nature of the family; care and support and distribution of care and support
**(b) Health**	Health costs and financing
**(c) Education and training**	Type of educational institution currently attending; level of educational attainment and field of educational attainment
**(d) Work**	N/A
**(e) Economic resources**	Income inequality; levels of remuneration; retirement income and access to particular services
**(f) Housing**	Home ownership and housing costs
**(g) Crime and justice**	N/A
**(h) Culture and leisure**	N/A
**Reference**	1. Australian Bureau of Statistics. 6540.0 - Microdata: Household Expenditure Survey and Survey of Income and Housing Canberra, Australia: Australian Bureau of Statistics; 2009-10 [01-04-2017]. Available from: http://bit.ly/2wYa5nq.<br>2. Australian Bureau of Statistics. 6540.0.00.001 - Household Expenditure Survey and Survey of Income and Housing - Confidentialised Unit Record Files, Technical Manual Canberra, Australia: Australian Bureau of Statistics; 2003-04 [01-04-2017]. Available from: http://bit.ly/2wZLhgr.

Table S11. Household Expenditure Survey, 1998-99 (Supplementary File, ID ABS: 96).

SURVEY CATEGORY	DETAIL
**Abbreviation**	HES
**Data type**	Sample data (nationally representative sample)
**Associated micro data**	1. Household Expenditure, including Fiscal Incidence Study (Supp. 1 ID ABS: 96)<br>*Data sets: Basic CURF*
**Aim of the data collection**	• The 1998-99 HES collected detailed information on expenditure, income and demographic characteristics
**How data was collected**	Collected information via face-to-face personal interviews
**Year(s) data collected**	1998-99
**Geographic coverage**	Whole of Australia (all states and territories) excluding non-private dwellings and very remote areas of Australia.
**Sample size**	*HES & SIH 1998-99*<br>Household records, n=6,892 private dwellings
**Wellbeing areas of concern**	Data available for key social issues for each area of concern
**(a) Family and community**	Changing nature of the family; care and support and distribution of care and support
**(b) Health**	Health costs and financing
**(c) Education and training**	Type of educational institution currently attending; level of educational attainment and field of educational attainment
**(d) Work**	N/A
**(e) Economic resources**	Income inequality; levels of remuneration; retirement income and access to particular services
**(f) Housing**	Home ownership and housing costs
**(g) Crime and justice**	N/A
**(h) Culture and leisure**	N/A
**Reference**	1. Australian Bureau of Statistics. 6544.0 - Technical Manual: Household Expenditure Survey, Basic CURF Canberra, Australia: Australian Bureau of Statistics; 1998-99 [01-04-2017]. Available from: http://bit.ly/2h2Gemc.

Table S12. Survey of Income and Housing, 1999-00, 2000-01, 2002-03, 2005-06, 2007-08, 2011-12, 2013-14 (Supplementary File, ID ABS: 101-106, 112).

SURVEY CATEGORY	DETAIL
**Abbreviation**	SIH
**Data type**	Sample data (nationally representative sample)
**Associated micro data**	1. Income and Housing (Supp. 1 ID ABS: 101)<br>*Data sets:* Basic CURF<br>*Data series:* Expanded CURF<br>2. Income and Housing (Supp. 1 ID ABS: 102)<br>*Data sets:* Basic CURF<br>*Data series:* Expanded CURF<br>3. Income and Housing (Supp. 1 ID ABS: 103)<br>*Data sets:* Basic CURF<br>*Data series:* Expanded CURF<br>4. Income and Housing (Supp. 1 ID ABS: 104)<br>*Data sets:* Basic CURF<br>*Data series:* Expanded CURF<br>5. Income and Housing (Supp. 1 ID ABS: 105)<br>*Data sets:* Basic CURF<br>*Data series:* Expanded CURF<br>6. Income and Housing (Supp. 1 ID ABS: 106)<br>*Data sets:* Basic CURF<br>*Data series:* Expanded CURF<br>7. Income and Housing Costs (Supp. 1 ID ABS: 112)<br>*Data sets:* Basic CURF<br>*Data series:* Expanded CURF
**Aim of the data collection**	• The survey provides detailed information about: demographic and socio-economic characteristics, including education and labour force status; income amounts and sources; assets and liabilities; housing characteristics, including tenure, dwelling type and housing costs; and child care use and costs.
**How data was collected**	Collected information via face-to-face personal interviews
**Year(s) data collected**	1999-00, 2000-01, 2002-03, 2005-06, 2007-08, 2011-12, 2013-14
**Geographic coverage**	Whole of Australia (all states and territories) excluding non-private dwellings and very remote areas of Australia.
**Sample size**	*SIH 1999-00*<br>Household records, n= 6,637 private dwellings<br>Person records, n=13,070 persons 15 years old and over
	SIH 2000-01<br>Household records, n= 6,786 private dwellings<br>Person records, n= 13,193 persons 15 years old and over
	SIH 2002-03<br>Household records, n=10,211 private dwellings<br>Person records, n=19,402 persons 15 years old and over
	SIH 2005-06<br>Household records, n= 9,961 private dwellings<br>Person records, n=19,208 persons 15 years old and over
	SIH 2007-08<br>Household records, n=9,345 private dwellings
	SIH 2011-12<br>Household records, n=14,569 private dwellings
	SIH 2013-14<br>Household records, n= 9,345 private dwellings

SURVEY CATEGORY	DETAIL
**Wellbeing areas of concern**	Data available for key social issues for each area of concern
**(a) Family and community**	Changing nature of the family; care and support and distribution of care and support
**(b) Health**	Health costs and financing
**(c) Education and training**	Type of educational institution currently attending; level of educational attainment; field of educational attainment
**(d) Work**	N/A
**(e) Economic resources**	Income inequality; levels of remuneration; retirement income and access to particular services
**(f) Housing**	Home ownership and housing costs
**(g) Crime and justice**	N/A
**(h) Culture and leisure**	N/A
**Reference**	1. Australian Bureau of Statistics. 6541.0.30.001 - Microdata: Income and Housing Canberra, Australia: Australian Bureau of Statistics; 2013-14 [01-04-2017]. Available from: http://bit.ly/2fgToM1.<br>2. Australian Bureau of Statistics. 6541.0.30.001 - Microdata: Income and Housing Canberra, Australia: Australian Bureau of Statistics; 2011-12 [01-04-2017]. Available from: http://bit.ly/2f4qDWf.<br>3. Australian Bureau of Statistics. 6541.0 - Technical Manual: Income and Housing, Basic and Expanded CURF Canberra, Australia Australian Bureau of Statistics; 2007-08 [01-04-2017]. Available from: http://bit.ly/2f44pUd.<br>4. Australian Bureau of Statistics. 6541.0 - Survey of Income and Housing - Confidentialised Unit Record File, Technical Manual Canberra, Australia: Australian Bureau of Statistics; 2005-06 [01-04-2017]. Available from: http://bit.ly/2eUA1I7.<br>5. Australian Bureau of Statistics. 6541.0 - Survey of Income and Housing - Confidentialised Unit Record File, Technical Paper (Reissue) Canberra, Australia: Australian Bureau of Statistics; 2002-03 [01-04-2017]. Available from: http://bit.ly/2wVWiQJ.<br>6. Australian Bureau of Statistics. 6541.0 - Technical Manual: Survey of Income and Housing, Expanded CURF Canberra, Australia: Australian Bureau of Statistics; 2000-01 [01-04-2017]. Available from: http://bit.ly/2w6eWFM.<br>7. Australian Bureau of Statistics. 6541.0.30.001 - Income and Housing Costs Survey Basic Canberra, Australia: Australian Bureau of Statistics; 1999-2000 [01-04-2017]. Available from: http://bit.ly/2jlL4PQ.

Table S13. National Aboriginal and Torres Strait Islander Social Survey, 2002, 2008, 2014-15 (Supplementary File, ID ABS: 151-153).

SURVEY CATEGORY	DETAIL
**Abbreviation**	NATSISS
**Data type**	Sample data (nationally representative sample)
**Associated micro data**	1. National Aboriginal and Torres Strait Islander Social Survey (Supp. 1 ID ABS: 151)<br>*Data series:* Expanded CURF, Table Builder<br>2. National Aboriginal and Torres Strait Islander Social Survey (Supp. 1 ID ABS: 152)<br>*Data series:* Expanded CURF<br>3. National Aboriginal and Torres Strait Islander Social Survey(Supp. 1 ID ABS: 153)<br>*Data series:* Expanded CURF
**Aim of the data collection**	• Provide broad information across key areas of social concern for Indigenous Australians aged 15 years or over, including information not previously available at the national, state/territory and broad regional levels<br>• Allow for inter-relationships between different areas of social concern to be explored and provide insight into the extent to which people face multiple social disadvantage<br>• Provide comparisons with results for the non-Indigenous population from the 2002 GSS and other surveys<br>• Measure selected changes over time
**How data was collected**	Collected information via face-to-face personal interviews
**Year(s) data collected**	2002, 2008, 2014-15
**Geographic coverage**	Whole of Australia (all states and territories) including remote areas in Australia but excluding non-private dwellings.
**Sample size**	*NATSISS 2002*<br>Household records, n= 5,887 private dwellings<br>Person records, n= 9,359 Indigenous persons 15 years old and over
	NATSISS 2008<br>Person records, n= 13,307 Indigenous persons 15 years old and over
	NATSISS 2014-15<br>Household records, n= 6,611 private dwellings<br>Person records, n= 11,178 Indigenous persons 15 years old and over
**Wellbeing areas of concern**	Data available for key social issues for each area of concern
**(a) Family and community**	Changing nature of the family; family dysfunction; care and support; distribution of care and support; voluntary work
**(b) Health**	Promotion and prevention
**(c) Education and training**	Type of educational institution currently attending; level of educational attainment; field of educational attainment; vocational vs. generalist education
**(d) Work**	Hours of work; work and training; unemployment; long term unemployment
**(e) Economic resources**	Income inequality; levels of remuneration; access to particular resources
**(f) Housing**	Home ownership; housing costs; access to housing
**(g) Crime and justice**	Levels and trends of crime
**(h) Culture and leisure**	Culture and leisure outcomes; availability of leisure time; cultural rights
**Reference**	1. Australian Bureau of Statistics. 4720.0.55.002 - Microdata: National Aboriginal and Torres Strait Islander Social Survey Canberra, Australia: Australian Bureau of Statistics; 2014-15 [01-04-2017]. Available from: http://bit.ly/2eUrqFg.

SURVEY CATEGORY	DETAIL
	2. Australian Bureau of Statistics. 4720.0 - National Aboriginal and Torres Strait Islander Social Survey: Users' Guide Canberra, Australia: Australian Bureau of Statistics; 2008 [01-04-2017]. Available from: http://bit.ly/2xuXxad.<br>3. Australian Bureau of Statistics. 4720.0 - National Aboriginal and Torres Strait Islander Social Survey: Expanded Confidentialised Unit Record File, Technical Paper Canberra, Australia: Australian Bureau of Statistics; 2002 [01-04-2017]. Available from: http://bit.ly/2fgV741.

Table S14a. Australian Health Survey (National Health Survey), 2001, 2004-05, 2007-08, 2011-12, and 2014-15 (Supplementary File, ID ABS: 155-160).

SURVEY CATEGORY	DETAIL
**Abbreviation**	AHS (NHS)
**Data type**	Sample data (nationally representative sample)
**Associated micro data**	1. National Health Survey (Supp. 1 ID ABS: 155)<br>*Data sets:* Basic CURF<br>*Data series:* Expanded CURF, Table Builder<br>2. Australian Health Survey, National Health Survey (Supp. 1 ID ABS: 156)<br>*Data sets:* Basic CURF<br>*Data series:* Expanded CURF, Table Builder<br>3. Australian Health Survey, Core Content - Risk Factors and Selected Health Conditions (Supp. 1 ID ABS: 157)<br>*Data series:* Expanded CURF, Table Builder<br>4. National Health Survey (Supp. 1 ID ABS: 158)<br>*Data sets:* Basic CURF<br>*Data series:* Expanded CURF<br>5. National Health Survey (Supp. 1 ID ABS: 159)<br>*Data sets:* Basic CURF<br>*Data series:* Expanded CURF<br>6. National Health Survey (Supp. 1 ID ABS: 160)<br>*Data sets:* Basic CURF<br>*Data series:* Expanded CURF
**Aim of the data collection**	The survey collected information about:<br>• The health status of the population, including long-term medical conditions experienced and recent injuries<br>• Use of health services such as consultations with doctors and dentists, visits to hospital and other actions people have recently taken for their health<br>• Health-related aspects of people's lifestyle, such as smoking, exercise and alcohol consumption<br>• Demographic and socio-economic characteristics.
**How data was collected**	Collected information via face-to-face personal interviews
**Year(s) data collected**	2001, 2004-05, 2007-08, 2011-12, and 2014-15
**Geographic coverage**	Whole of Australia (all states and territories) while very Remote areas of Australia and discrete Aboriginal and Torres Strait Islander communities were excluded
**Sample size**	*NHS 2001*<br>Household records, n= 17,918 private dwellings<br>Person records, n= 26,862 persons
	NHS 2004-05<br>Household records, n=19,501 private dwellings<br>Person records, n=25,906 persons (19,501 adult and 6,405 child)
	NHS 2007-08<br>Household records, n=15,787 private dwellings
	NHS 2011-12<br>Household records, n=15,565 private dwellings<br>Person records, n=20,426 persons
	AHS 2014-15<br>Household records, n=14,726 private dwellings<br>Person records, n=19,257
**Wellbeing areas of concern**	Data available for key social issues for each area of concern
**(a) Family and community**	Changing nature of the family

SURVEY CATEGORY	DETAIL
**(b) Health**	Aging of the population; promotion and prevention; national health priority areas; socioeconomic inequalities; health costs and financing; workplace health and safety
**(c) Education and training**	Type of educational institution currently attending; level of educational attainment; field of educational attainment
**(d) Work**	Hours of work; unemployment; long term unemployment
**(e) Economic resources**	Income equality; levels of remuneration; access to particular services
**(f) Housing**	Home ownership; housing costs
**(g) Crime and justice**	N/A
**(h) Culture and leisure**	N/A
**Reference**	1. Australian Bureau of Statistics. 4324.0.55.001 - Microdata: National Health Survey Canberra, Australia: Australian Bureau of Statistics; 2014-15 [01-04-2017]. Available from: http://bit.ly/2xwNIs2.<br>2. Australian Bureau of Statistics. 4324.0.55.001 - Microdata: Australian Health Survey, National Health Survey Canberra, Australia: Australian Bureau of Statistics; 2011-12 [01-04-2017]. Available from: http://bit.ly/2fgg7b9.<br>3. Australian Bureau of Statistics. 4324.0.55.003 - Microdata: Australian Health Survey, Core Content - Risk Factors and Selected Health Conditions Canberra, Australia: Australian Bureau of Statistics; 2011-12 [01-04-2017]. Available from: http://bit.ly/2eU91sb.<br>4. Australian Bureau of Statistics. 4324.0 - Information Paper: National Health Survey, Basic and Expanded CURF Canberra, Australia: Australian Bureau of Statistics; 2007-08 [01-04-2017]. Available from: http://bit.ly/2jlY1ZQ.<br>5. Australian Bureau of Statistics. 4324.0 - Information Paper: National Health Survey - Confidentialised Unit Record Files Canberra, Australia: Australian Bureau of Statistics; 2004-05 [01-04-2017]. Available from: http://bit.ly/2w6wTUw.<br>6. Australian bureau of Statistics. 4324.0 - Information Paper: National Health Survey - Confidentialised Unit Record Files Canberra, Australia: Australian bureau of Statistics; 2001 [01-04-2017]. Available from: http://bit.ly/2wnUknE.

Table S14b. National Nutrition and Physical Activity Survey, 2011-12 (Supplementary File, ID ABS: 165, 166).

SURVEY CATEGORY	DETAIL
**Abbreviation**	NNPAS
**Data type**	Sample data (nationally representative sample)
**Associated micro data**	1. Australian Health Survey, Nutrition and Physical Activity (Supp. 1 ID ABS: 165)<br>*Data sets:* Basic CURF<br>*Data series:* Expanded CURF, Table Builder<br>2. Australian Health Survey, Core Content - Risk Factors and Selected Health Conditions (Supp. 1 ID ABS: 166)<br>*Data series:* Expanded CURF, Table Builder
**Aim of the data collection**	• NHS was designed to collect a range of information from Australians about health related issues, including: health status, risk factors, socioeconomic circumstances, health-related actions and use of medical services<br>• The NNPAS is a component survey of the broader AHS
**How data was collected**	Collected information via face-to-face personal interviews and telephone interviews
**Year(s) data collected**	2011-12
**Geographic coverage**	Whole of Australia (all states and territories) while very remote areas of Australia and discrete Aboriginal and Torres Strait Islander communities were excluded
**Sample size**	*NNPAS*<br>Household records, n=9,519 private dwellings<br>Person records, n=12,153
	NNPAS CATI<br>Person records, n=7,735
	NNPAS Pedometer<br>Person records, n= 6,061
	Combined NHS/NNPAS (the Core)<br>Household records, n= 25,080 private dwellings<br>Person records, n= 31,837
**Wellbeing areas of concern**	Data available for key social issues for each area of concern
**(a) Family and community**	N/A
**(b) Health**	Promotion and prevention; national health priority areas; socioeconomic inequalities
**(c) Education and training**	Type of educational institution currently attending; level of educational attainment; field of educational attainment
**(d) Work**	N/A
**(e) Economic resources**	N/A
**(f) Housing**	N/A
**(g) Crime and justice**	N/A
**(h) Culture and leisure**	Culture and leisure outcomes; availability of leisure time
**Reference**	1. Australian Bureau of Statistics. 4324.0.55.002 - Microdata: Australian Health Survey: Nutrition and Physical Activity Canberra, Australia: Australian Bureau of Statistics; 2011-12 [01-04-2017]. Available from: http://bit.ly/2jkRRZO.<br>2. Australian Bureau of Statistics. 4324.0.55.003 - Microdata: Australian Health Survey, Core Content - Risk Factors and Selected Health Conditions Canberra, Australia: Australian Bureau of Statistics; 2011-12 [01-04-2017]. Available from: http://bit.ly/2eU91sb.

Table S15. Adult Literacy and Life Skills Survey, 2006 (Supplementary File, ID ABS: 176).

SURVEY CATEGORY	DETAIL
**Abbreviation**	ALLS
**Data type**	Sample data (nationally representative sample)
**Associated micro data**	1. Adult Literacy and Life Skills (Supp. 1 ID ABS: 176)<br>*Data sets:* Basic CURF<br>*Data series:* Expanded CURF
**Aim of the data collection**	• The ALLS is designed to identify and measure literacy, numeracy and problem-solving skills, which can be linked to social and economic characteristics both across and within countries.<br>• An additional literacy measure, health literacy, is also available.<br>• The key objectives of the survey are to profile the distribution of prose literacy, document literacy, numeracy, analytic reasoning and health literacy in the adult population (15 to 74 years), and to identify sub populations whose performance in these skill domains may place them at risk.
**How data was collected**	Collected information via face-to-face personal interviews
**Year(s) data collected**	2006
**Geographic coverage**	Whole of Australia (all states and territories) excluding non-private dwellings and very remote areas of Australia.
**Sample size**	Household records, n= 8,988 private dwellings
**Wellbeing areas of concern**	Data available for key social issues for each area of concern
**(a) Family and community**	N/A
**(b) Health**	National health priority areas;
**(c) Education and training**	Type of educational institution currently attending; level of education attainment; field of education attainment; literacy and numeracy
**(d) Work**	Hours of work; unemployment; long term unemployment
**(e) Economic resources**	Income inequality; levels of remuneration; access to particular services
**(f) Housing**	N/A
**(g) Crime and justice**	N/A
**(h) Culture and leisure**	N/A
**Reference**	1. Australian Bureau of Statistics. 4228.0.30.001 - Microdata: Adult Literacy and Life Skills Survey, Australia, (Second Edition) Canberra, Australia: Australian Bureau of Statistics; 2006 [01-04-2017]. Available from: http://bit.ly/2wZPZe8.

Table S16. Vocational Education and Training in Schools, 2006 and 2011 (Supplementary File, ID ABS: 191).

SURVEY CATEGORY	DETAIL
**Abbreviation**	VET
**Data type**	Sample data (nationally representative sample and integrated data set)
**Associated micro data**	1. Outcomes from Vocational Education and Training in Schools (Supp. 1 ID ABS: 191)
**Aim of the data collection**	• Integrated data set comprising of the 2006 Vocational Education and Training in Schools and 2011 Census of Population and Housing Integrated Dataset.<br>• Aims to provide a dataset to analyse the post school labour market and education outcomes of participants in Vocational Education and Training in Schools.<br>• Persons aged 15-19 years who were enrolled in a VET in Schools module or unit of competency in 2006 and who also responded to the 2011 Census.
**How data was collected**	*VET 2006*<br>Data on VET in Schools are collected from the administrative records of enrolments at registered training organisations held by senior secondary assessment authorities in each state and territory submitted to the data to the National Centre for Vocational Education Research<br>*CPH 2011*<br>Collected by the delivery and re-collection of a paper survey
**Year(s) data collected**	2006 and 2011
**Geographic coverage**	Whole of Australia (all states and territories)
**Sample size**	n=84,412 (50.52% (84,412 out of 167,088) of the in-scope VET in Schools records were successfully linked to Census records.
**Wellbeing areas of concern**	Data available for key social issues for each area of concern
**(a) Family and community**	Care and support; voluntary work
**(b) Health**	N/A
**(a) Education and training**	Type of educational institution currently attending; level of educational attainment; field of educational attainment
**(b) Work**	Hours of work; unemployment; long term unemployment
**(c) Economic resources**	Income inequality; levels of remuneration
**(d) Housing**	Home ownership; housing costs
**(e) Crime and justice**	N/A
**(f) Culture and leisure**	N/A
**Reference**	1. Australian Bureau of Statistics. 4260.0.55.001 - Microdata: Outcomes from Vocational Education and Training in Schools Canberra, Australia: Australian Bureau of Statistics; 2006-2011 [01-04-2017]. Available from: http://bit.ly/2wVR3QG.

Table S17. Household, Income and Labour Dynamics in Australia Survey (Supplementary File, ID LONG: 299).

SURVEY CATEGORY	DETAIL
**Abbreviation**	HILDA
**Data type**	Longitudinal data
**Associated micro data**	1. Household, Income and Labour Dynamics in Australia Survey (Supp. 1 Long ID: 299)
**Aim of the data collection**	• Primary objective is to support research questions falling within three broad and inter-related areas of income, labour market and family dynamics.
**How data is collected**	Collected information via face-to-face and telephone personal interviews
**Year(s) data collected**	2001-present
**Geographic coverage**	Whole of Australia (all states and territories) excluding diplomatic personnel of overseas governments, overseas residents in Australia, people living in remote areas, or members of non-Australian defence forces
**Waves**	15
**Cohorts**	2
**Sample size**	Original sample, n=13,753<br>Top up, n=3853
**Wellbeing categories**	Data available for key social issues for each area of concern
**(a) Family and community**	Changing nature of the family; family dysfunction; care and support; distribution of care and support; voluntary work
**(b) Health**	Aging population; preventable deaths; promotion and prevention; national health priority areas; socioeconomic inequalities; health costs and financing;
**(c) Education and training**	Type of educational institution currently attending; level of educational attainment; field of education attainment
**(d) Work**	Hours worked; work and training; unemployment; long term unemployment; long term unemployment; unpaid household work; voluntary work
**(e) Economic resources**	Income inequality; level of remuneration; retirement income; access to particular services
**(f) Housing**	Home ownership; housing costs; access to housing; community housing
**(g) Crime and justice**	N/A
**(h) Culture and leisure**	N/A
**Reference**	1. The University of Melbourne. HILDA Survey Melbourne, Australia: The University of Melbourne; 2017 [01-04-2017]. Available from: http://melbourneinstitute.unimelb.edu.au/hilda.

Table S18. Longitudinal Surveys of Australian Youth (Supplementary File, ID LONG: 301).

SURVEY CATEGORY	DETAIL
**Abbreviation**	LSAY
**Data type**	Longitudinal data
**Associated micro data**	1. Longitudinal Surveys of Australian Youth (Supp. 1 LONG ID: 301)
**Aim of the data collection**	• LSAY track young people as they move from school into further study, work, and other destinations<br>• LSAY provides a source of information about young people and their pathways, helping researchers and policymakers make educated decisions about youth policies<br>• Survey participants enter the study when they are about 15 years old and are contacted once a year until they are 25
**How data is collected**	Collect information by web-based surveys or telephone interview
**Year(s) data collected**	1995-present
**Geographic coverage**	Nationally representative sample of whole of Australia (all states and territories)
**Waves**	12
**Cohorts**	6
**Sample size**	Y95, n=13,613<br>Y98, n=14,117<br>Y03, n=10,370<br>Y06, n=14,170<br>Y09, n=14,251<br>Y15, n=unavailable at time of scoping review
**Wellbeing categories**	Data available for key social issues for each area of concern
**(a) Family and community**	Volunteer work
**(b) Health**	National health priority areas;
**(c) Education and training**	Type of educational institution currently attending; level of educational attainment; field of educational attainment; barriers to education and training; literacy and numeracy
**(d) Work**	Hours of work; work and training; unemployment; voluntary work
**(e) Economic resources**	Income inequality; levels of remuneration; access to particular services
**(f) Housing**	Home ownership; housing costs
**(g) Crime and justice**	N/A
**(h) Culture and leisure**	Culture and leisure outcomes; availability of leisure time
**Reference**	1. National Centre for Vocational Education Research. The Longitudinal Surveys of Australian Youth Australia: Commonwealth of Australia; 2017 [01-04-2017]. Available from: https://www.lsay.edu.au/.

Table S19. Australian Census Longitudinal Dataset (Supplementary File, ID LONG: 306).

SURVEY CATEGORY	DETAIL
**Abbreviation**	ACLD
**Data type**	Longitudinal data
**Associated micro data**	1. Australian Census Longitudinal Dataset (Supp. 1 LONG ID: 306)
**Aim of the data collection**	• ACLD comprises a 5% sample from the 2006 Census with records from the 2011 for exploring how Australian society is changing over time
**How data is collected**	Collected by the delivery and re-collection of a paper survey
**Year(s) data collected**	2006 and 2011
**Geographic coverage**	Whole of Australia (all states and territories)
**Waves**	2
**Cohorts**	1
**Sample size**	n=800,759 (82% of the 979,661 sample records from 2006 were linked to a 2011 Census record)
**Wellbeing categories**	Data available for key social issues for each area of concern
**(a) Family and community**	Changing nature of the family; care and support; distribution of care and support; voluntary work; wellbeing of regional and local area communities
**(b) Health**	N/A
**(c) Education and training**	Type of educational institution currently attending; level of educational attainment; field of educational attainment
**(d) Work**	Hours of work; work and training; unemployment; long term unemployment and voluntary work
**(e) Economic resources**	Income inequality; level of remuneration
**(f) Housing**	Home ownership; housing costs; access to housing; and community housing
**(g) Crime and justice**	N/A
**(h) Culture and leisure**	N/A
**Reference**	1. Australian Bureau of Statistics. 2080.0 - Microdata: Australian Census Longitudinal Dataset Canberra, Australia: Australian Bureau of Statistics; 2006-2011 [01-04-2017]. Available from: http://bit.ly/2f57WS9.

Table S20. Longitudinal Labour Force Survey (Supplementary File, ID LONG: 307).

SURVEY CATEGORY	DETAIL
**Abbreviation**	LLFS
**Data type**	Longitudinal data
**Associated micro data**	1. Longitudinal Labour Force Survey (Supp. 1 LONG ID: 307)
**Aim of the data collection**	• To provide data on labour force status, employment status, hours worked, underemployment status, industry and occupation or main/last job, income and earnings, unemployment status, duration of unemployment, not in the labour force status, retirement status, as well as relevant standard demographic, family, educational, and other details and characteristics.
**How data is collected**	The first interview is conducted face-to-face. Subsequent interviews are conducted by telephone.
**Year(s) data collected**	2008-2010
**Geographic coverage**	Whole of Australia (all states and territories) excluding non-private dwellings and very remote areas of Australia.
**Waves**	8
**Cohorts**	1
**Sample size**	Household records, n=~29,000 private dwellings with one-eighth of the sample being replaced each month
**Wellbeing categories**	Data available for key social issues for each area of concern
**(a) Family and community**	Changing nature of the family
**(b) Health**	N/A
**(c) Education and training**	Type of educational institution currently attending; level of educational attainment; field of educational attainment
**(d) Work**	Hours of work; unemployment; long term unemployment
**(e) Economic resources**	Income inequality; levels of remuneration; retirement income
**(f) Housing**	Home ownership; housing costs
**(g) Crime and justice**	N/A
**(h) Culture and leisure**	N/A
**Reference**	1. Australian Bureau of Statistics. 6602.0 - Microdata: Longitudinal Labour Force Canberra, Australia: Australian Bureau of Statistics; 2008-10 [01-04-2017]. Available from: http://bit.ly/2xjwAFA.

Table S21. Personality & Total Health Through Life (Supplementary File, ID LONG: 309).

SURVEY CATEGORY	DETAIL
**Abbreviation**	PATH
**Data type**	Longitudinal data
**Associated micro data**	1. Personality & Total Health Through Life (Supp. 1 LONG ID: 309)
**Aim of the data collection**	Original aims include:<br>• To delineate the course of depression, anxiety, substance use and cognitive ability with increasing age across the adult life span.<br>• To identify environmental risk, genetic risk and protective factors influencing individual differences in the course of these characteristics.<br>• To investigate interrelationships over time between the three domains of: depression and anxiety, substance use, and cognitive ability and dementia.<br>Additional aims include (clinical outcomes that constitute the major burden of disease within the Australian community):<br>• Infertility, fertility and pregnancy changes in family structure, relationship formation and separation, menopause and retirement.
**How data is collected**	Waves 1-3 face-to-face in person interview<br>Wave 4 online questionnaire
**Year(s) data collected**	1999-present
**Geographic coverage**	Randomly sampled from the electoral roll of the Australian Capital Territory and the nearby city of Queanbeyan.
**Waves**	4
**Cohorts**	1
**Sample size**	n= 7,485 comprising young (aged 20–24 at baseline), midlife (aged 40–44 at baseline) and older (aged 60–64 at baseline) adults
**Wellbeing categories**	Data available for key social issues for each area of concern
**(a) Family and community**	Changing nature of the family; family dysfunction; care and support
**(b) Health**	Ageing of the population; promotion and prevention; national health priority areas; socioeconomic inequalities
**(c) Education and training**	Type of educational institution currently attending; level of educational attainment; field of educational attainment
**(d) Work**	Unemployment; unpaid household work; voluntary work
**(e) Economic resources**	N/A
**(f) Housing**	Housing costs
**(g) Crime and justice**	N/A
**(h) Culture and leisure**	Culture and leisure outcomes; availability of leisure time
**Reference**	1. Centre for Research on Ageing HW. Personality & Total Health (PATH) Through Life: Australian National University; 2017 [01-04-2017]. Available from: http://crahw.anu.edu.au/research/projects/personality-total-health-path-through-life.

Table S22. Australian Temperament Project (Supplementary File, ID LONG: 311).

SURVEY CATEGORY	DETAIL
**Abbreviation**	ATP
**Data type**	Longitudinal data
**Associated micro data**	1. Australian Temperament Project (Supp. 1 LONG ID: 311)
**Aim of the data collection**	• The study aims to trace the pathways to psychosocial adjustment and maladjustment across the lifespan, and to investigate the contribution of personal, family and environmental factors to development and wellbeing.
**How data is collected**	Mail out questionnaire
**Year(s) data collected**	1983-present
**Geographic coverage**	Urban and rural areas of Victoria
**Waves**	16
**Cohorts**	1
**Sample size**	Original sample, n=2,443
**Wellbeing categories**	Data available for key social issues for each area of concern
**(a) Family and community**	Changing nature of the family; family dysfunction; care and support
**(b) Health**	Promotion and prevention; national health priority areas
**(c) Education and training**	Type of educational institution currently attending; level of educational attainment; field of educational attainment; literacy and numeracy
**(d) Work**	Career pathways
**(e) Economic resources**	N/A
**(f) Housing**	N/A
**(g) Crime and justice**	N/A
**(h) Culture and leisure**	N/A
**Reference**	1. Studies. AIoF. Australian Temperament Project Canberra, Australia: Australian Government; 2017 [01-04-2017]. Available from: http://www3.aifs.gov.au/atp/.
